# Congenital Lobar Emphysema in Early Adulthood

**DOI:** 10.7759/cureus.12590

**Published:** 2021-01-09

**Authors:** Sara Dâmaso, Nuno R Carreira, Catarina Gonçalves, Patrício Aguiar

**Affiliations:** 1 Serviço de Medicina 2, Hospital de Santa Maria, Centro Hospitalar Universitário Lisboa Norte, Lisboa, PRT; 2 Serviço de Medicina 1, Hospital de Santa Maria, Centro Hospitalar Universitário Lisboa Norte, Lisboa, PRT

**Keywords:** congenital lobar emphysema, congenital disease, hyperlucent lung, emergency radiology

## Abstract

Congenital lobar emphysema (CLE) is a rare developmental abnormality of the lower respiratory tract. This disease is caused by cartilage or connective tissue defects, leading to overdistention of a pulmonary lobe. CLE is mainly diagnosed in early childhood, though it might be rarely found in young adults. Due to its rarity, it can be misdiagnosed with other conditions. Here we report a case of a previously healthy young female complaining of dyspnea and thoracic pain after a commercial flight. Physical and radiological examinations were consistent with the diagnosis of CLE.

## Introduction

Congenital lobar emphysema (CLE), also known as congenital alveolar overdistension, congenital hyperlucent lobe or congenital lobar overinﬂation, is a developmental anomaly of the lower respiratory tract caused by defects in cartilage or connective tissue [1]. This condition affects males and females in a ratio of 3:1 and it has an estimated prevalence worldwide of about 1 in 20,000 to 1 in 30,000 live births [2,3]. CLE is characterized by the presence of a valve mechanism that causes airflow obstruction. The consequent air trapping leads to overdistention of one or more lobes [4]. The diagnosis is usually early in life, with 50% of patients diagnosed by four to six weeks of age. Less than 5% of patients are diagnosed after the age of six months [5]. However, cases of CLE diagnosed in early adulthood have been reported [5]. This paper reports a rare case of CLE that only became symptomatic in young adulthood.

## Case presentation

**Figure 1:**
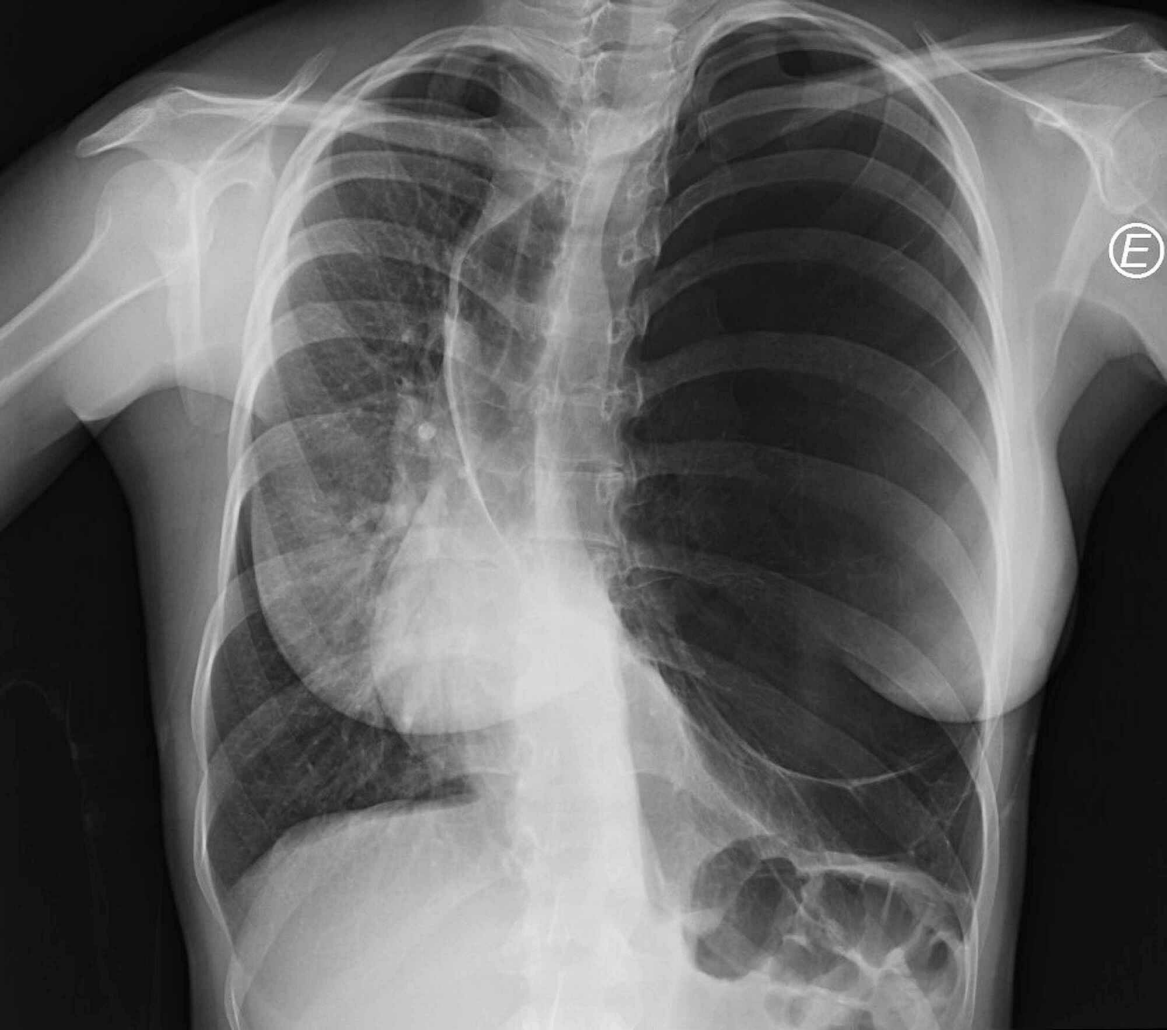
Chest radiograph.

A 20-year-old German Caucasian female was admitted to the ED with complaints of dyspnea at rest and anterior thoracic pain. These symptoms started during a commercial flight, initially with enough severity to justify assistance in the airport after landing and emergent transportation to our hospital. She had no previous medical history except for an episode of bronchiolitis during childhood. No smoking, alcoholic or drug abuse habits were reported. On admission, the patient was already asymptomatic. Blood pressure, cardiac frequency and peripheral oxygen saturation were normal without supplemental therapy. On physical examination, a thoracic asymmetry was visible. Breath sounds were abolished in the left hemithorax and hyperresonance was noted at thoracic percussion. Laboratory evaluation including complete blood count, C-reactive protein, D-dimers, creatine kinase, lactate dehydrogenase, troponin and arterial blood gas test (ABG) were unremarkable. Chest X-ray showed an hyperlucent left lung which was initially suspected to be an hypertensive pneumothorax (Figure 1).

**Figure 2:**
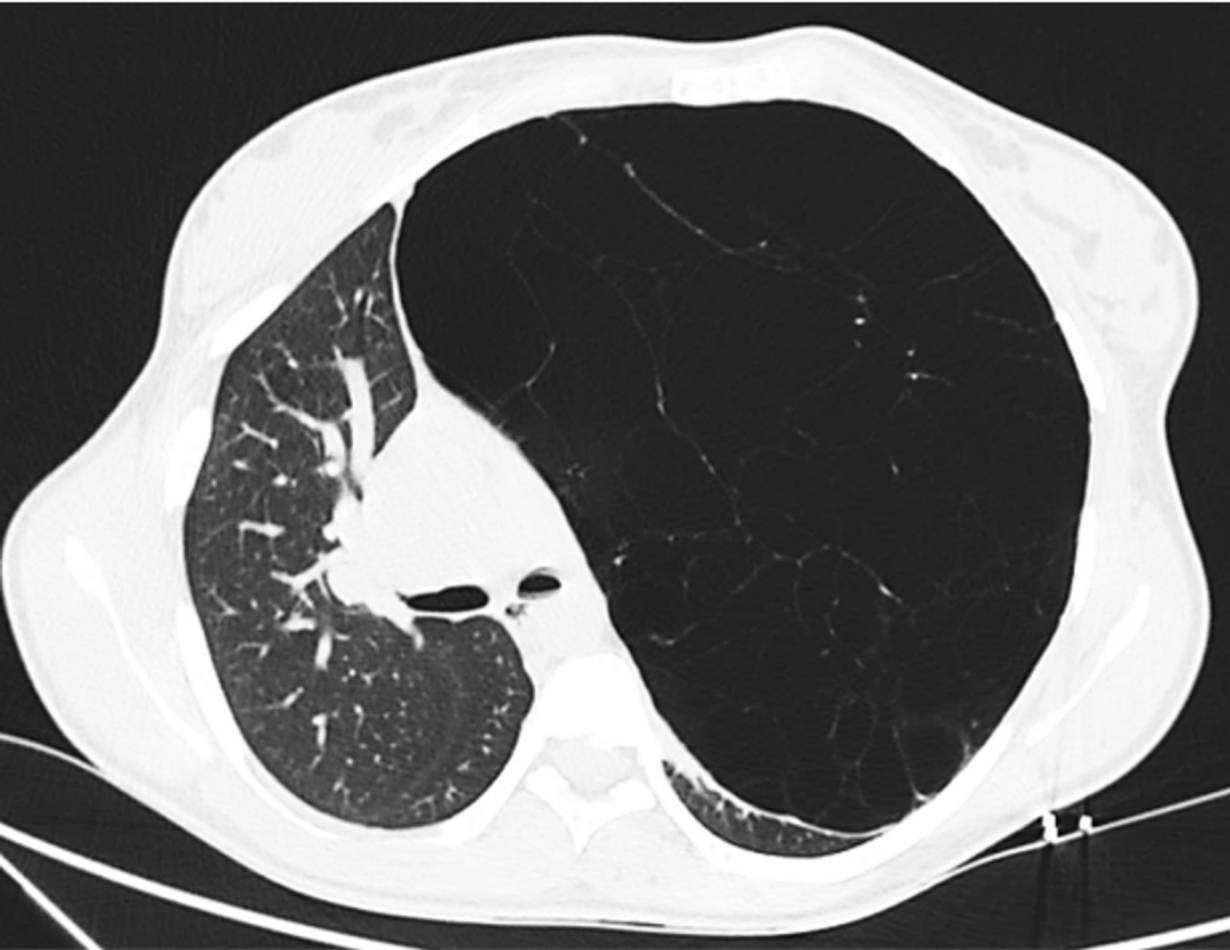
Chest computed tomography (axial).

**Figure 3:**
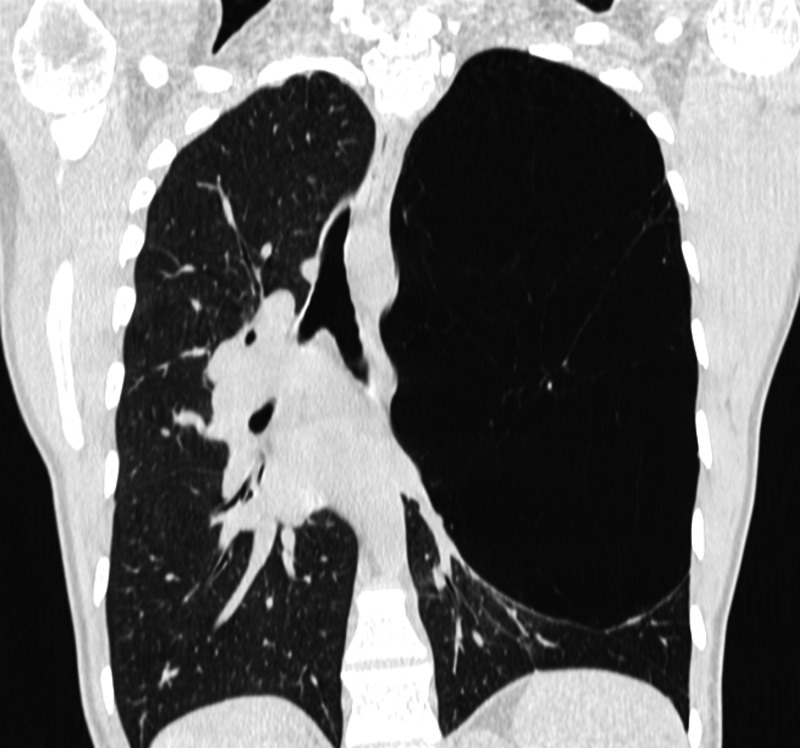
Chest computed tomography (coronal).

Complete remission of symptoms, absence of hemodynamic instability, and ABG findings were, however, not suggestive of that hypothesis. A thoracic computed tomography (CT) was performed and described an area of hyperinflation occupying the upper two-thirds of the left lung (23 cm in the longest axis) with scarce vasculature, causing contralateral mediastinal shift and compressive atelectasis of the remaining pulmonary parenchyma, suggestive of congenital lobar emphysema (Figures 2, 3).

Based on clinical presentation and radiologic findings, a diagnosis of CLE was established and the patient was admitted to the medical ward for clinical surveillance. Afterwards, in the absence of symptoms that would justify immediate surgical management, a conservative approach was chosen.

## Discussion

CLE is a congenital development defect of the lower respiratory tract characterized by overinflation of a pulmonary lobe. The most common forms arise in the left upper lobe (43%) followed by the right middle lobe (32%) [1]. An outflow obstruction causes the affected lobe to become overinﬂated with impaired ventilation/perfusion. Involvement of multiple lobes is highly uncommon [6]. Approximately 30% of cases are symptomatic at birth and the majority by the age of 12 months [7]. Rarely CLE is diagnosed in adult patients who did not experience any symptoms during childhood [6].

The etiology is unknown in about 50% of CLE patients [1]. The most frequently identified cause of CLE is obstruction of the developing airway (25% of cases). Other identified causes of CLE are summarized in Table 1. 

**Table 1:** Identified mechanisms of congenital lobar emphysema (adapted from Demir et al. 2019 [1]).

Identified mechanisms of congenital lobar emphysema	
1. Bronchial disease	Bronchial stenosis
	Bronchial atresia
	Bronchomalacia
2. Vascular abnormalities	Pulmonary arterial sling anomalies
	Abnormal pulmonary venous return
3. Extrinsic compression	Bronchogenic cysts
	Mediastinal tumors

Signs and symptoms include tachypnea, wheezing, chronic cough and recurrent respiratory tract infections [1,6]. Physical examination reveals impaired thoracic expansion and use of accessory respiratory muscles, vocal fremitus and hyperresonance in the affected lobe area at thoracic percussion and diminished or abolished breath sounds at pulmonary auscultation [1]. Progressive overinﬂation over time can cause compression in the adjacent organs and displacement of mediastinal structures.

CLE is mainly a radiologic diagnosis. A posteroanterior chest X-ray should be the initial workup in patients with respiratory symptoms. Hyperlucency of the affected lobe can be seen accompanied by atelectasis of adjacent lobes and mediastinal shift to the opposite side [8]. Nonetheless, chest CT scan remains the gold standard for the diagnosis of CLE, as it allows evaluation of adjacent lobes and contralateral lung. Contrast-enhanced CT is used to exclude vascular anomalies and mediastinal masses [9]. Removal of foreign bodies and search for anatomical variations might be done through bronchoscopy [10]. Ventilation-perfusion scan shows the characteristic decrease in ventilation and perfusion in the affected lobe [9]. Prenatal diagnosis can sometimes be made through ultrasonography; however, since CLE requires ventilation to be clinically evident, most cases are diagnosed after birth [11]. Echocardiographic evaluation is also necessary in these patients since CLE is accompanied by cardiac abnormalities in about 14% to 20% of cases [1].

The standard treatment for severe cases of CLE is lobectomy through thoracotomy [12]. In adults, the use of thoracoscopic approaches is increasing and may be considered. In cases mild symptomatic or asymptomatic where the patient is clinically stable, conservative treatment is a recommended option [1]. These patients should be followed closely due to potential clinical progression and future need for surgical intervention [12,13].

## Conclusions

The purpose of this report is to highlight a rare clinical entity with an uncommon late presentation. Only a few case reports of CLE in adults are described in the literature and differential diagnosis should be carefully assessed with most common lung diseases in adults. Its misdiagnosis may lead to unnecessary and even harmful procedures. In our case, the misinterpretation of the initial radiological findings with a hypertensive pneumothorax could lead to a needle thoracostomy approach. In this setting, this procedure might cause severe complications for the patient, for instance a permanent bronchopleural fistula. Finally, these patients must be managed according to radiological features and symptom severity.
